# Antioxidants in Oak (*Quercus* sp.): Potential Application to Reduce Oxidative Rancidity in Foods

**DOI:** 10.3390/antiox12040861

**Published:** 2023-04-02

**Authors:** Elsa Daniela Othón-Díaz, Jorge O. Fimbres-García, Marcela Flores-Sauceda, Brenda A. Silva-Espinoza, Leticia X. López-Martínez, Ariadna T. Bernal-Mercado, Jesus F. Ayala-Zavala

**Affiliations:** 1 Centro de Investigación en Alimentación y Desarrollo, A.C, Carretera Gustavo Enrique Astiazarán Rosas 46, Hermosillo 83304, Sonora, Mexico; 2 Departamento de Investigación y Posgrado en Alimentos, Universidad de Sonora, Blvd. Luis Encinas y Rosales S/N, Col. Centro, Hermosillo 83000, Sonora, Mexico

**Keywords:** *Quercus*, antioxidants, phenolic compounds, food rancidity, lipid oxidation

## Abstract

This review explores the antioxidant properties of oak (*Quercus* sp.) extracts and their potential application in preventing oxidative rancidity in food products. Oxidative rancidity negatively impacts food quality, causing changes in color, odor, and flavor and reducing the shelf life of products. The use of natural antioxidants from plant sources, such as oak extracts, has gained increasing interest due to potential health concerns associated with synthetic antioxidants. Oak extracts contain various antioxidant compounds, including phenolic acids, flavonoids, and tannins, which contribute to their antioxidative capacity. This review discusses the chemical composition of oak extracts, their antioxidative activity in different food systems, and the safety and potential challenges related to their application in food preservation. The potential benefits and limitations of using oak extracts as an alternative to synthetic antioxidants are highlighted, and future research directions to optimize their application and determine their safety for human consumption are suggested.

## 1. Introduction

Food rancidity is defined as food spoilage due to the oxidation of lipids, mainly unsaturated fatty acids, which results in the formation of off-flavors, off-odors, and harmful compounds. This process is characterized by the breakdown of fats and oils into fatty acids and other oxidation products responsible for the negative impact of rancidity [1,2]. Lipid oxidation can occur during raw material handling, processing, and storage [3]. The main effects of lipid oxidation on food are changes in color and texture and the appearance of rancid tastes and odors, decreasing shelf life and causing consumer rejection [4,5]. In addition, advanced lipid oxidation end products (ALEs) can affect human health, being implicated in diseases such as arteriosclerosis, cancer, inflammation, and aging processes, among others [4,6]. Moreover, lipid oxidation and rancidity represent a problem in the food industry, as they are directly involved in increased food waste and economic losses [3], so it is necessary to find safe solutions to lessen the damage they cause.

Food antioxidant additives can counteract the adverse effects caused by lipid oxidation. An antioxidant is defined as any substance that can significantly delay or prevent substrate oxidation at low concentrations [7]. A wide variety of antioxidants are used to prevent food from spoiling, which can be synthetic or natural [8,9]. Synthetic additives have been widely used since their appearance due to their low cost, high purity, and constant activity at low concentrations; however, some harmful effects have been reported in animals [8,9,10]. It has been pointed out that synthetic antioxidants such as butyl hydroxyanisole (BHA) and butylated hydroxytoluene (BHT) could have carcinogenic properties in addition to increasing the risk of allergies and causing poisoning and metabolic disorders when consumed in high doses [9,11]. In addition, its use in foods for infants and children has been prohibited, and antioxidants such as BHA have been banned in certain countries, such as the European Union and Japan [8,11,12]. For this reason, efforts have focused on the search for new antioxidant compounds that can be added to foods without causing harm to consumers.

A proposed strategy to reduce food rancidity is using extracts from safe and effective natural sources with antioxidant potential. Specifically, extracts from oak (*Quercus* sp.) are an alternative against oxidative rancidity that present antioxidant phenolic compounds [13]. Some studies have reported positive results by adding extracts from different oak species to meat products. For example, chicken breasts treated with *Q. suber* extract inhibited lipid oxidation by up to 97.7% [14], while *Q. ilex*-treated chicken patties had an inhibition of ~75% during their cooking, chilled, and reheated process [15]. Pork patties treated with *Q. alba* showed inhibition values of 97.1% [16]. In all the previous cases, adding the *Quercus* extract to foods significantly decreased lipid oxidation compared to untreated samples. Other foods added with oak extracts are pasteurized milk (*Q. infectoria*) [17], soybean oil (*Q. branti*) [18], sunflower oil, and orange juice (*Q. ballota*) [19]. Even when these results are promissory to reduce food waste and assure safety against rancidity, still more knowledge can be generated regarding its efficacy in other susceptible food systems and at different stages of the supply chain (i.e., raw material handling, processing, or storage). In addition, this review describes how determining the optimal concentration of oak extracts for specific food products can be used to achieve maximum antioxidant activity while minimizing any potential adverse impacts on flavor, odor, and appearance.

The antioxidant activity of the *Quercus* species has been associated with the high presence of bioactive compounds, such as polyphenols [20]. Among the main compounds reported are gallic acid, ellagic acid, vanillic acid, syringic acid, ferulic acid, quercetin, kaempferol, catechin, epicatechin, caffeic acid, and others [21,22]. The primary mechanism of action of these compounds is radical scavenging activity, showing a high inhibition of radicals such as 1,1-diphenyl-2-picrylhydrazyl (DPPH), 2,20-azinobis (3-ethylbenzothizoline- 6-sulfonic acid) diammonium salt (ABTS), and the hydroxyl radical (•OH) [23]. Likewise, it has been reported that extracts of *Q. salicina* rich in phenolic compounds increased the activity of superoxide dismutase (SOD) and catalase (CAT), two important enzymes for antioxidant defense in cells exposed to oxygen [23,24]. However, a lack of knowledge was detected regarding the potential synergistic effects of oak extract compounds with other natural or synthetic antioxidants. Similarly, it is suggested to optimize the extraction procedures and perform a deep molecular characterization in the extracts. Finally, it is recommended to guarantee the economic feasibility of incorporating oak extracts into food products and their potential impact on reducing food waste and economic losses. Therefore, this review aims to discuss the potential uses of oak extracts as a suitable additive to reduce oxidative rancidity in different food matrices.

## 2. Spoilage in Food: Oxidative Rancidity

### 2.1. Rancidity in Food: Economic and Health Consequences

Rancidity has become a challenge that compromises food security [5,25,26]. This phenomenon shortens the food product's shelf life, causes consumer rejection, economic losses, and food waste [3,5,26]. Even when no specific records of food waste caused by rancidity were found, the Food and Agriculture Organization of the United Nations (FAO) reports that approximately 1/3 of all food produced for human consumption, corresponding to 1300 million tons, is lost (decrease in edible food at production level) and wasted (discard of edible food at retail and consumer levels) by different causes during the production chain, which translates into 936 billion dollars per year [27,28,29]. Notably, 14% of food produced is lost during harvest and retail, while 17% of total production is wasted at home (11%), food service (5%), and retail trade (2%) [30]. 

**Figure 1:**
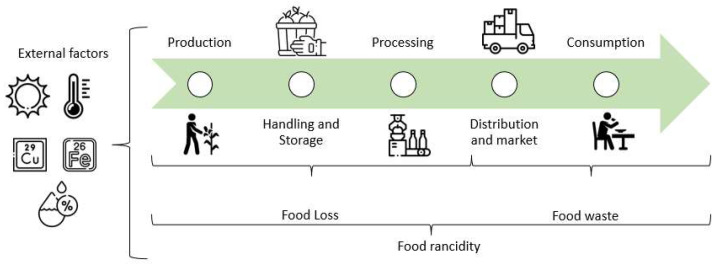
Environmental stressors triggering rancidity at different stages of food production.

Comparing developed and developing countries shows significant food loss and waste differences. In developed countries, the total amount is 56%, of which 21% is lost, and 35% is wasted. In the case of developing countries, the total is equivalent to 44%, where loss comprises 30%, and waste is 14% [29]. Meanwhile, about 20 million tons of food in Mexico are wasted annually, resulting in economic losses of 400 billion Mexican pesos [31]. Rancidity constitutes one of the causes that lead to food loss and waste, as it can occur during all stages of food production (handling, processing, and storage) (Figure 1) [4]; however, its real impact has not been quantified. To fully understand the impact of rancidity on food loss and waste, specific statistics need to be generated, including the amount of food lost and wasted by rancidity during harvest, processing, and storage [28]. During the production, handling, storage, processing, distribution and market, and home preparation stages of food, various conditions exist that can contribute to lipid oxidation and the development of rancidity [4]. In the early stages of production, high temperatures and exposure to light, air, and trace metals can initiate and accelerate lipid oxidation. During handling and storage, temperature fluctuations and exposure to light, air, and moisture can also contribute to lipid oxidation, while, in processing, high heat and pressure treatment, exposure to oxygen, and the addition of prooxidants such as iron and copper can also lead to lipid oxidation. During distribution and market, improper storage, light exposure, and high temperatures can also contribute to rancidity. Finally, during home preparation, excessive heating and inappropriate cookware can also lead to this problem [4,32].

Foods highly susceptible to lipid oxidation include oils and fats, fatty fish, nuts and seeds, dairy products, and meat [4,25,33,34]. Additionally, it would be useful to analyze the number of consumers that reject food products specifically due to rancidity and the reasons behind the rejection. The monetary loss due to rancidity can also be determined by considering the production cost, disposal expenses, and potential sale loss. Finally, a data comparison of lost and wasted food due to rancidity in different regions can provide specific knowledge and support the search for tailored solutions [28,35,36].

The rancidity process causes negative changes in foods at a nutritional level. Free radicals, lipid oxidation products involved in rancidity, cause vitamin degradation and changes in protein functionality [4,37]. Other compounds resulting from oxidation, such as lipid peroxides, can decompose fat-soluble vitamins, such as vitamins E, A, and their provitamins. These vitamins act as natural antioxidants, when they react with free radicals, they protect food from oxidation and decrease their antioxidant activity [38]. Similarly, during this process, the loss of long-chain n-3 polyunsaturated fatty acids (PUFA n-3) also occurs, directly affecting the nutritional properties of the food since they are essential for humans [5,39]. Future research in this area could focus on quantifying the content of specific vitamin and nutrient degradation in specific food products due to rancidity. Additionally, it would be valuable to study the extent of the loss of essential fatty acids (PUFA n-3) in food products and how this affects their nutritional quality. Furthermore, research could aim to develop preservation techniques to reduce the negative impact of rancidity on the nutritional quality of food products, as well as alternative methods to replace lost nutrients. Additionally, studies on the long-term effects of consuming food products with decreased nutritional quality due to rancidity could provide important information for public health. 

The consumption of rancid products can be dangerous due to their health risks. Some compounds produced by lipid oxidation, such as malondialdehyde, acrolein, 4-hydroxy-trans-nonenal, 4-hydroxy-trans-hexanal, and crotonaldehyde-like compounds, are implicated in the pathogenesis of many diseases such as inflammatory processes, aging, cancer, arteriosclerosis, and Alzheimer’s disease [1,3,6]. Lipid hydroperoxides contribute to cell cytotoxicity, while aldehydes and oxysterols have proinflammatory, cytotoxic, and mutagenic effects [4]. ALEs have been considered to cause inflammation, fibrosis, and atypical cell proliferation [6]. However, more research is needed to establish the relationship between the consumption of rancid food, the level of toxic by-products, and the development of diseases. Additionally, research could be conducted to determine the most effective methods to reduce or eliminate these health risks, such as better food storage techniques or adding natural antioxidants to prevent rancidity.

In the case of oils oxidized by heating, their consumption could increase the risk of cardiovascular disease, endothelial malfunction, lipid peroxidation, oxidative stress, genotoxicity, carcinogenicity, and reduced glucose uptake. In addition, the excessive generation of free radicals is known to cause liver damage, resulting in diseases such as hepatitis, cirrhosis, and liver tumors [40]. Moreover, the formation of trans fatty acids in reused oils is harmful because it decreases good cholesterol (HDL) and increases bad cholesterol (LDL), as well as increases triglyceride concentrations and the risk of suffering a heart attack and developing cancer [41]. In fact, the toxicological and pathogenic properties that could result from the ingestion of lipid oxidation products, such as aldehydes contained in cooking oils heated according to standard frying practices, influence the potential development of cardiovascular diseases, carcinogenic properties, contribution to neurodegenerative disorders, hypertensive effects, development of diabetes, and respiratory and pulmonary complications, especially in the case of acrolein [42]. Future research could be suggested to investigate the health effects of oils oxidized by heating, such as the link between the consumption of these oils and cardiovascular disease, liver damage, and various diseases such as diabetes and cancer. Further research could also examine the role of trans fatty acids in heart disease and the potential for developing new frying practices that can reduce the formation of harmful compounds. Additionally, research could be done to understand the mechanisms behind the toxicological and pathogenic properties of lipid oxidation products and how to mitigate them in cooking oils.

Several studies have been able to prove the consequences of the consumption of compounds from lipid oxidation. Chen et al. [43] showed in a rat model that exposure to acrolein affects muscle regeneration. In addition, muscle mass loss was promoted at a concentration of 2.5 and 5 mg/kg for 4 weeks, and weight decreased significantly. Meanwhile, Chung and collaborators [44] found that administering crotonaldehyde to F344 rats at low concentrations (0.60 mmol/L) for 113 weeks resulted in an 87% incidence of liver tumors. High doses (6.00 mmol/L) caused severe liver damage in 43% of the treated rats, while the remaining 57% developed abnormal cell foci. Other studies have indicated that other factors, such as inhalation of volatile aldehydes and other carbonyl compounds from cooking oil fumes by workers in poorly ventilated fast food/restaurant establishments, are also considered to be a major threat to human health, especially since it is associated with a high incidence of lung cancer [42]. Given the effects on health caused by consuming these compounds, it is important to know the mechanism that leads to the appearance of rancidity in foods to reduce it and avoid affecting the consumer’s health.

### 2.2. Rancidity Process: Lipid Oxidation and Hydrolysis

**Figure 2:**
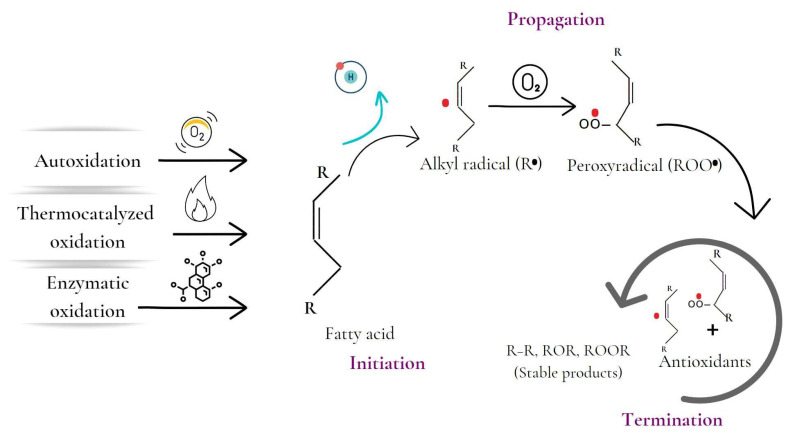
Stages of the lipid oxidation process initiated by autocatalyzed, thermocatalyzed, and enzymatic oxidation mechanisms.

Lipid oxidation has been described through several mechanisms, such as autocatalyzed, thermocatalyzed, enzymatic, and photo-oxidation (Figure 2) [3]. Autoxidation is the most significant process in lipid oxidation and causes oxidative rancidity. The process involves the interaction of unsaturated fatty acids with oxygen, leading to a continuous chain reaction of free radicals [1,3]. The process has three phases: initiation, propagation, and termination. During the initiation phase, hydrogen is extracted from an unsaturated fatty acid, forming an alkyl radical. The propagation phase involves the magnification of radical production, while the termination phase involves the reaction of the radicals with each other or with antioxidants, becoming relatively stable [4]. Autoxidation is influenced by internal and external factors such as fatty acid profile, temperature, light, and prooxidants such as transition metals [3].

Foods rich in unsaturated fatty acids, especially polyunsaturated fatty acids (PUFAs), are more susceptible to lipid autooxidation [3,4,37]. This is because unsaturated fatty acids are more reactive to oxygen, leading to oxidative rancidity [45]. Examples of foods rich in unsaturated fatty acids and susceptible to lipid autooxidation include oils, nuts, seeds, and fatty fish [25]. Additionally, foods with high-fat content exposed to air, light, and heat are more susceptible to lipid autoxidation [1,4].

Thermocatalyzed lipid oxidation refers to the process triggered by the application of heat. This type significantly contributes to food spoilage and rancidity [3,46]. During this process, the interaction of unsaturated fatty acids with heat can activate transition metals such as iron and copper, causing the initiation of oxidative reactions [47]. The thermocatalyzed oxidation mechanism also includes the three phases of initiation, propagation, and termination, where free radicals are generated and multiplied and then become non-reactive compounds [46]. Other factors that can influence the rate of thermocatalyzed oxidation include pro-oxidants, trace metals, and the fatty acid profile of the lipid in question [46,47]. Foods more susceptible to suffering from thermocatalyzed lipid oxidation contain high levels of unsaturated fatty acids and are exposed to high temperatures, such as fried and grilled foods, processed foods, baked goods, and snacks [48]. Additionally, the presence of metals such as iron and copper and pro-oxidants can contribute to the oxidation process and increase the risk of lipid oxidation in these foods [46].

Enzymatic lipid oxidation is another process that occurs due to the activity of lipoxygenases (LOXs) and dioxygenases, acting mainly on polyunsaturated fatty acids [49]. The oxidation of fatty acids by these enzymes generates hydroperoxides via free radical mechanisms, which, in turn, are converted into secondary oxidation products such as aliphatic aldehydes, alcohols, ketones, and esters by a series of complex reactions [50,51]. These reactions contribute to the lipid oxidation of food, mainly meat products, seeds, and oils, causing it to become rancid [49,51,52]. Factors that affect enzymatic oxidation are the content of lipoxygenases in the food, the form in which iron is found in the active site of the enzyme (ferrous form), and external factors such as singlet oxygen, light, metal ions, and radiation [4,50,52]. It is important to control the enzymatic oxidation process to ensure the quality and safety of food products.

Another process that leads to lipid oxidation and results in rancidity is photo-oxidation [3]. During this process, hydroperoxides are formed thanks to the presence of sensitizers and exposure to light [4]. The reactions carried out during photo-oxidation are divided into three pathways. First, singlet oxygen is formed through the reaction of an excited triplet sensitizer with molecular oxygen; when it reacts with unsaturated fatty acids, it forms hydroperoxides without forming alkyl radicals. In the second case, a reaction between the excited sensitizer and triplet oxygen produces a superoxide radical anion that can react with unsaturated fatty acids and initiate lipid oxidation. This reaction is carried out in the presence of metals. As for the third path, the excited triplet sensitizer reacts with an unsaturated fatty acid, producing an alkyl radical that can subsequently initiate the free radical chain reaction mechanism, producing lipid oxidation [4]. 

The lipid photo-oxidation process is considered to be faster than auto-oxidation [3]. Among the factors that affect photo-oxidation are the light exposure time of food, the presence of photosensitizers, the most common being chlorophyll, hemeproteins, porphyrins, and riboflavin, and the presence of metal prooxidants [4,53]. The molecules degraded by photo-oxidation are proteins, fats and oils, pigments, and vitamins, with milk, meat products, vegetable oils, and wines being the most affected food products [54]. Additionally, foods stored in transparent containers or exposed to light during processing and storage are more susceptible to lipid photo-oxidation [53].

**Figure 3:**
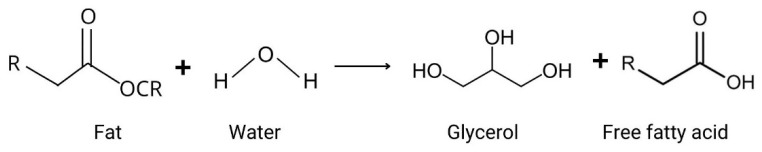
Fat hydrolysis reaction.

In addition to the different mechanisms of lipid oxidation, there is another chemical reaction that causes rancidity in food: hydrolysis (Figure 3). Hydrolytic rancidity occurs when lipids are broken down into free fatty acids, glycerol, and other compounds through the action of lipase enzymes, heat, or moisture [25]. This process can lead to off-flavors and odors in food products, negatively impacting their quality. Factors such as high temperatures and extreme pH accelerate hydrolysis; lipases act on acylglycerol at different degrees of specificity [55]. Hydrolytic rancidity is particularly relevant in high-fat food products where lipase activity is present or where moisture content and temperature conditions favor the hydrolysis of lipids, including in fried foods, vegetable oils (e.g., soybean, corn, and sunflower oil), milk, butter, and meat [25,55,56]. Although this review focuses on oxidative rancidity, it is important to acknowledge hydrolytic rancidity as another significant cause of food spoilage. 

Further research on lipid oxidation and hydrolytic rancidity could deepen understanding and improve food quality and safety. This can involve studying the initiation of autoxidation, the impact of internal and external factors, pro-oxidants, antioxidants, and the effects of different forms of lipid oxidation such as thermocatalyzed, enzymatic, and photo-oxidation in specific food systems. Areas of interest include understanding the reaction between polyunsaturated fatty acids and oxygen and the role of pro-oxidants and evaluating the impact of thermocatalyzed, enzymatic, photo-oxidation, and hydrolysis on food quality and safety, especially new products.

### 2.3. The Impact of Rancidity on Food Frying Oils: Associated Health Risks and Food Waste

Frying is a cooking method that many cultures worldwide have used for centuries. Some cultures have a deep history of using frying for food preparation, with different regional variations and techniques. For example, in Europe, fried dishes such as French fries and fish and chips are stapled foods, while, in Asian countries such as Japan and China, tempura and stir-fry are popular dishes. In the Middle East, falafel and baklava are examples of fried foods enjoyed for generations. In Mexico, frying is a traditional cooking method for preparing various dishes, such as tacos, sopes, corn tortilla chips, and churros. Mexican cuisine has a rich tradition of using frying for food preparation, and it continues to be a popular cooking method in Mexican homes and restaurants [57,58,59,60]. The relevance of frying in Mexican cuisine highlights the importance of this cooking method in preserving culinary traditions. There are several methods, including deep-frying, where food is immersed in a bath of hot fat or oil; *sautéing*, in which a small amount of fat or oil is used in a frying pan; and *roasting*, in which protein-rich food is prepared in an oven or griddle using minimal addition of fat or oil [61,62]. This cooking method imparts desirable characteristics to the food, increasing its palatability due to fat absorption, crust formation, and pleasant flavors and odors [63]. However, food frying oils are highly susceptible to lipid oxidation due to different factors.

During frying, the fats or oils reach temperatures between 160 and 180 °C, even up to more, depending on the type of frying [61]. In addition, it is common for oils to be reused at the household and commercial levels to minimize costs and maximize profits [40]. Frying oils, such as soybean, canola, and corn, contain high levels of polyunsaturated fatty acids. Continued use of oil in these conditions brings negative consequences to the oil, starting with changes in its physical appearance, such as increased viscosity and darkening. Similarly, chemical changes occur, including oxidation, hydrolysis, and polymerization, where the fried food absorbs many of the resulting oxidative products, such as hydroperoxides and aldehydes, affecting its flavor and color. Other by-products produced by this cooking method include alcohols, cyclic compounds, polymers, dimers, and free fatty acids [63,64]. Additionally, the presence of light, air, and pro-oxidants such as iron and copper in fried food can accelerate the oxidation rate. Furthermore, storing used frying oils for a long period, even in cool and dark conditions, can increase the likelihood of oxidation, affecting the quality and safety of the fried food.

The deterioration of oils starts from processing and storage, where autoxidation and photo-oxidation processes can occur [65]. Then, during frying, hydrolysis of the oil occurs, where fatty acids are released due to high temperatures and moisture from the food. This process leads to the appearance of “soapy” flavors and a decrease in the oil’s smoke point, referring to the temperature at which it starts to burn and degrade [25,37,62]. Similarly, lipid oxidation continues generating volatile and non-volatile compounds such as esters, aldehydes, ketones, and peroxides, one of the significant deteriorating reactions in oils [62,63]. 

Preventing rancidity in food frying oils is of utmost importance to the food industry and consumers’ health. For the food industry, maintaining the quality and shelf life of their products are crucial to the business success. Rancid oils not only negatively impact the taste and aroma of the food, but also reduce its nutritional value and potentially introduce harmful compounds into the food. On the other hand, for consumers, consuming rancid oils can have adverse health effects, such as an increased risk of oxidative stress and chronic diseases. By preventing rancidity in food frying oils, the food industry can provide high-quality and safe products while preserving the flavors associated with traditional cuisine.

## 3. The Advantages of Natural Antioxidants for Reducing Lipid Rancidity

Natural antioxidants have been widely used in food preservation due to their unique properties that offer many advantages over synthetic molecules. Unlike synthetic agents, natural options derived from plants, fruits, and spices have been proven to be safe and effective in reducing lipid rancidity [66]. These antioxidants possess a combination of beneficial compounds that act synergistically to delay oxidative processes, improving the shelf life and quality of food products [67]. Additionally, natural antioxidants are typically recognized as safe by regulatory agencies and are favored by consumers who prefer natural food additives [7]. By incorporating natural antioxidants, the food industry can not only improve food quality and safety, but also appeal to the growing demand for natural and organic food products [67].

### 3.1. Balancing Quality and Safety: The Debate Between Natural and Synthetic Antioxidants

Natural antioxidants, such as vitamins, minerals, and plant extracts, are derived from natural sources and are commonly used in food applications [66]. On the other hand, synthetic antioxidants are chemically synthesized and generally considered more potent than natural antioxidants, but they also come with potential health concerns [68]. Both antioxidants protect food from oxidative rancidity, which can cause off-flavors, reduce nutrient content, and increase the risk of harmful oxidation by-products [26].

Food antioxidants can be added during all production steps, and their use is strictly controlled [7,11,68]. The two main regulatory organizations for these substances are the European Food Safety Authority (EFSA) in the European Union and the Food and Drug Administration (FDA) in the United States. Other regulatory bodies are the Food and Agriculture Organization (FAO), World Health Organization (WHO), Expert Committee on Food Additives, and Codex Alimentarius [7]. In Mexico, the agency in charge of regulating the use of additives is the Federal Commission for the Protection against Health Risks (COFEPRIS) [69].

According to its functions, the Codex Alimentarius has classified food additives into 27 families, while EFSA has organized them into 9 “E numbers.” Within these classifications, antioxidant agents constitute one of the most important families because oxidation is one of the main causes of food degradation [11]. Antioxidants, classified within the E300–E399 number block, are used to extend the shelf life of foods by preventing rancidity, loss of color, development of odors, and loss of texture, among other undesirable effects [7]. Table 1 lists some antioxidants approved by the EFSA, their maximum dosage range, and reported adverse reactions.

In the particular case of antioxidant additives, there are several ways to classify them. According to their origin, they can be classified as natural, natural-identical, which correspond to compounds chemically synthesized to mimic natural ones, and synthetic, equivalent to molecules that do not exist in nature [8]. Generally, natural antioxidants are added to meat, fish, nuts, vegetables, fruits, beverages, and canned food, while synthetic ones are added to oils, cheeses, and chips [9]. It should be noted that there is still no consensus on this classification, so, officially, all antioxidants are still considered in a single group [7]. Primary antioxidants and secondary or synergistic molecules represent another distinction. Primary antioxidants act by oxidizing themselves, which allows food components to remain unchanged, while synergistic antioxidants reinforce the action of the primary ones [8,11].

Antioxidants play a crucial role in preventing oxidative rancidity in food, thus extending its shelf life and preserving its quality. The classification of antioxidants into natural and synthetic, primary and secondary, and natural-identical and synthetic highlights the diversity and complexity of these compounds. While various international and national agencies strictly regulate their use, further research is still needed to fully understand the different modes of action of antioxidants and their effects on food quality and human health and identify the most effective and safe combinations of antioxidants for specific food applications. Additionally, it is important to consider the environmental impact of synthetic antioxidants and to explore the potential of natural and natural-identical molecules as sustainable alternatives. By addressing these gaps in knowledge, the food industry can ensure the safe and effective use of antioxidants in preserving food quality while protecting consumer health and the environment.

### 3.2. Health Problems Associated with Synthetic Antioxidants

The use of antioxidants in agri-food and food products has become common due to the economic benefits it brings by reducing losses due to rancidity. Wsowicz and collaborators [38] mentioned that some state governments have a financial and public health interest in the widespread use of these compounds since adding antioxidants in high-fat foods could influence consumer health. This action would lower the incidence of certain diseases related to oxidative stress and the public expenditure they represent. However, synthetic antioxidants have also been shown to have adverse health effects [68], leading to some consumers’ fear and rejection and promoting the search for natural options.

**Figure 4:**
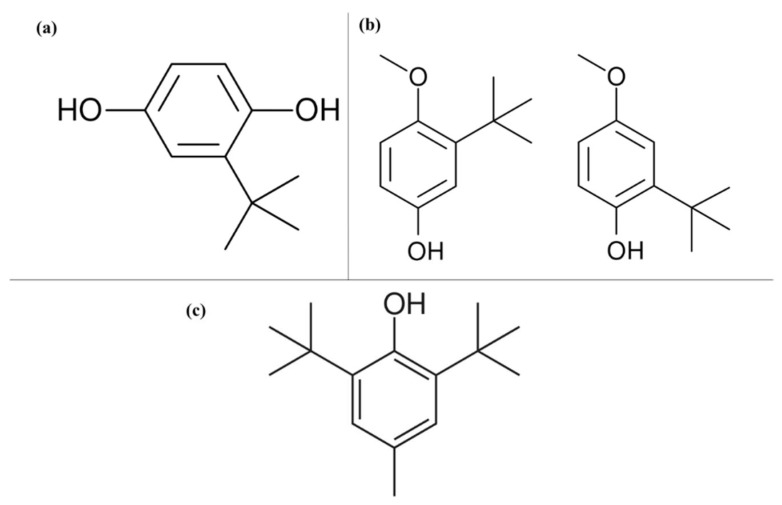
Chemical structures of the molecules. (**a**) Tert-butyl hydroquinone (TBHQ); (**b**) Butyl hydroxyanisole (BHA); (**c**) Butylated hydroxytoluene (BHT).

The synthetic compounds BHA and BHT (Figure 4) have been identified to possess carcinogenic properties at high doses; furthermore, prolonged exposure to BHT can cause chronic poisoning and metabolic disorders [9,11]. Similarly, manifestation and exacerbation of allergic reactions such as chronic urticaria, rash, angioedema, and atopic dermatitis have been reported (Table 1), as well as increased blood cholesterol and lipid levels. These undesired side effects have led to these compounds not being used in foods for infants and children [11,69,71]. The antioxidant TBHQ (Figure 2) has also been considered as carcinogenic and genotoxic, so its maximum permitted use limit does not exceed 200 mg/kg, while the use of antioxidants from gallates is also prohibited in foods for infants and children due to the risk of developing methemoglobinemia [11]. Other effects caused by this group of antioxidants are allergic reactions in asthmatic people and those who cannot tolerate acetylsalicylic acid [11]; propyl gallate can also cause apoptosis and DNA cleavage [9].

Whether to use synthetic or natural additives is still a hotly debated topic. It is considered that these antioxidants are better than synthetic ones because they come from a natural source; however, this statement is not always supported by scientific data [72]. This does not mean that the negative effects caused by synthetic antioxidants should be left aside. Hence, it is necessary to closely monitor the doses used in food and continue searching for new compounds that present less risk to human health. The potential public health benefits and financial savings of using antioxidants in high-fat foods make it an important area for future research. The challenge is finding a balance between the benefits of using antioxidants to extend the shelf life of food products and the potential health risks these compounds pose. It is also important to consider the varying conditions of use and the specific type of antioxidant when evaluating its effectiveness and safety.

## 4. Oak Extract as a Source of Natural Antioxidant Additives against Rancidity

### 4.1. Antioxidant Compounds Found in the Oak Tree

For centuries, oak extracts have been used as a natural antioxidant in food and beverage preservation. The tannins and other compounds in oak extracts have long been recognized for preventing oxidation [22]. The ancient Greeks and Romans used oak extracts in winemaking and food preservation, and the practice of aging wine in oak barrels has continued to this day [73]. The use of oak extracts as a natural antioxidant has been expanded beyond the wine industry, currently being proposed as an ingredient in a wide variety of foods and beverages, including meat, cheese, and tea [21]. The resurgence of interest in natural and healthy food additives has led to increased use of oak extracts as an alternative to synthetic antioxidants.

*Quercus* is a genus of evergreen or deciduous trees belonging to the Fagaceae family, composed of more than 500 species distributed throughout Europe, Asia, North Africa, and America [20,21]. They are very valuable due to their wood, fruits, and charcoal obtained from them and the medicinal properties in their bark, fruits, and leaves. For this reason, they have long been used in traditional medicine to treat conditions such as burns, hemorrhages, gastrointestinal diseases, dermatitis, and throat infections [21,23]. In the cooperage industry, they are also widely used to manufacture barrels to store wines, an important part of the wine maturation. Because they are economical, they have become one of the most profitable woods for this industry [13,21]. In Mexico, around 150 species have been reported, representing the second most important forest resource and used mainly for manufacturing handicrafts, firewood, and charcoal [74,75]. 

Recently, *Quercus* species have attracted researchers’ attention due to the bioactive compounds they might contain; hence, studies have been conducted to describe their chemical constituents, functional properties, and beneficial effects [23]. Extracts of oak leaves, branches, acorns, and bark have been evaluated in search of compounds with antioxidant, antitumor, anti-inflammatory, antidiabetic, hypocholesterolemia, antihypertensive, and antimicrobial activity, with promising results [13,20]. The main bioactive phytochemicals in *Quercus* are phenolic compounds, volatile organic compounds, vitamin E, sterols, aliphatic alcohols, and fatty acids [21]. 

**Figure 5:**
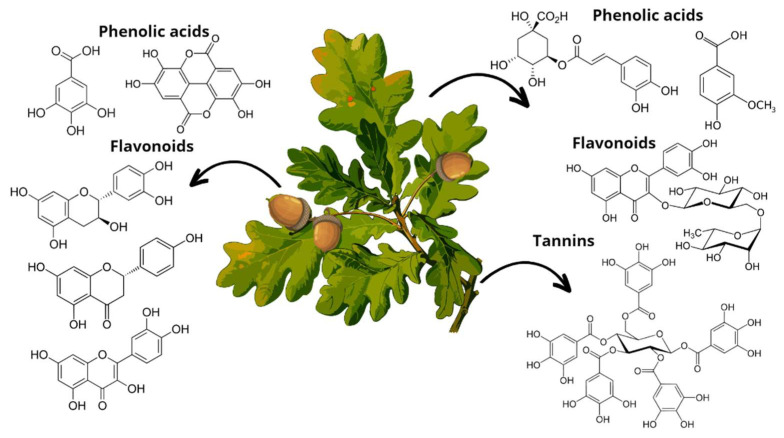
Main phenolic compounds found in *Quercus* leaves (chlorogenic acid, vanillic acid); acorns (gallic acid, ellagic acid, epicatechin, naringenin, quercetin), and bark (ellagitannins).

Specifically, phenolic compounds have been identified in all oak organs. Most flavonoid and non-flavonoid constituents are involved in phenylpropanoid intermediate metabolism via the shikimate pathway. The best-known are gallic acid, vanillic acid, syringic acid, ferulic acid, quercetin, kaempferol, catechin, and epicatechin [21]. Figure 5 lists some of the phenolic compounds found in each oak organ. External factors such as seasonal changes and the level of maturity of the evaluated parts of the oak influence its phytochemical composition, presenting variations in phenolic compounds and flavonoids [23]. It is worth mentioning that, among species, there are also complex variations in their chemical composition [21], which opens more possibilities to find new compounds with high bioactive potential.

As mentioned above, phenolic compounds are known for their high antioxidant capacity. Their structural arrangement confers a strong acidic characteristic to the phenol ring, responsible for its antioxidant power. For this reason, there is a strong correlation between the total polyphenol content and the extract's antioxidant activity [76]. In *Quercus*, gallic acid, ellagic acid, and ellagitannins such as castalagin, vescalagin, and roburin have been identified as potent antioxidant compounds, also presenting antimutagenic and anticarcinogenic activities derived from their antioxidant power [21]. Different in vivo and in vitro studies of the oak extract demonstrated a high antioxidant capacity linked to its high content of polyphenolic compounds (Table 2) [20]. Tuyen and collaborators [77] studied leaf and bark extracts of three oak species (*Q. crispula*, *Q. salicina,* and *Q. serrata*), analyzing them by DPPH and ABTS assays. The results showed high amounts of total phenolic compounds, being higher in leaf extracts. As for the antioxidant activity, a higher activity was found in the free phenol fraction of the leaf extracts. Among the predominant compounds found were ellagic, chlorogenic, and benzoic acids. The revised studies highlighted that most *Quercus* species have high antioxidant activity; however, they pointed out that leaf extract of *Q. salicina* had the highest potential, with stronger activity.

In another study, Gezici and Sekeroglu [78] studied the extracts of *Q. coccifera* acorns, whose parts are used for coffee brewing. The shelled acorn extract showed a higher total phenol content, while the shell extract had the highest flavonoid content. Regarding antioxidant activity, DPPH and Ferric Reducing Antioxidant Power (FRAP) assays showed remarkable activity in all extracts, highlighting the acorn cup extract at a concentration of 1000 µg/mL, with 91% radical scavenging over DPPH. Pinto and collaborators [79] analyzed extracts from *Q. cerris*, resulting in a total phenol content of 2070 mg GAE/L, while the total flavonoids were 285.3 mg CE (Catechin Equivalent)/L. In addition, the extract presented high efficiency in terms of reactive oxygen species (ROS) and nitrogen (RNS) scavenging activity associated with ellagic acid and derivatives of gallotannins and ellagitannins. In Mexico, Valencia-Avilés and collaborators [13] studied the bark extracts of *Q. laurina*, *Q. crassifolia,* and *Q. scytophylla* with two extraction methods, finding that *Q. crassifolia* hot water extract possessed the highest concentration of polyphenols and the best ROS scavenging capacity. 

Alañón and collaborators [80] studied the methanolic extracts of the wood of the oaks most used in cooperage (*Q. robur*, *Q. petraea,* and *Q. pyrenaica*). The results showed that *Q. robur* had the highest phenolic content and therefore a higher antioxidant activity. Among the phenolic compounds identified in the three species were gallic acid, protocatechuic acid, *p*-coumaric acid, ellagic acid, and ellagitannins. On the other hand, Soriano et al. [16] analyzed the aqueous extracts of the wood of *Q. alba*, another important oak within the cooperage industry. The extract showed a high phenolic content and remarkable antioxidant activity against DPPH and ABTS radicals. Volatile compounds were analyzed, finding benzenic compounds, lactones, and furanic compounds. Likewise, other oak species studied that have shown good results in terms of phenolic content and antioxidant activity are *Q. suber* [14], *Q. ilex* [15], and *Q. branti* [18], whose extracts have been proposed as antioxidant additives in foods given their antioxidant potential.

In summary, the study of extracts from various *Quercus* species has yielded promising results as a potential source of antioxidant compounds varying in chemical composition among species. Despite the numerous studies that have been carried out, there are still many species to be covered, as well as giving more importance to the antimicrobial, antitumor, and anti-inflammatory potential, among others, that these compounds present. The research on oak extracts and *Quercus* species has shown promising results for their potential as natural antioxidants, anti-inflammatory, and antimicrobial agents [13,20,77]. The main bioactive phytochemicals in the *Quercus* species include phenolic compounds, volatile organic compounds, vitamin E, sterols, aliphatic alcohols, and fatty acids. Phenolic compounds are the most abundant and have been found to have high antioxidant capacity, with gallic acid, ellagic acid, and ellagitannins identified as potent antioxidant compounds [21]. In vitro and in vivo studies of oak extracts have demonstrated high antioxidant capacity, with variations in their potency depending on the species, part of the oak used, and external factors such as seasonal changes and level of maturity [13,43,75,77]. Further research can be conducted to explore the chemical composition of different *Quercus* species to determine the best oak parts for extraction and to investigate the functional properties and potential health benefits of these extracts when applied in food systems.

### 4.2. Food Application of Oak Extracts

Given the high antioxidant potential of *Quercus* species extracts, some researchers have shown interest in using their compounds as antioxidant additives in processed foods. The ability of different oak species extracts to inhibit lipid oxidation that causes oxidative rancidity in various foods, mainly meat products, has been studied (Table 3). Lavado and collaborators [14] studied the ability of *Q. suber* leaf extracts to control oxidation in cooked chicken breasts stored under refrigeration. It was found that after 10 days of storage, the extract prevented lipid oxidation by up to 97.7%, reducing thiobarbituric acid reactive substances with a capacity equivalent to the synthetic additive BHT. In another study, *Q. alba* wood extract [16] was tested as a natural preservative of refrigerated pork patties for 12 days. The results showed that the patties treated with the extract presented higher antioxidant capacity, a 97.1% reduction in lipid oxidation, and a decrease in generating volatile compound products of oxidation reactions compared to sodium ascorbate as synthetic control. 

Ferreira and collaborators [15] studied *Q. ilex* acorn extract on cooked, stored, and reheated chicken patties. Samples containing the extract maintained a low number of thiobarbituric acid-reactive substances and lipid-derived volatiles throughout processing (0.2 mg TBARS/kg). In addition to meat foods, other food products have been evaluated with oak extracts. For example, the antioxidant capacity of *Q. branti* acorn extract on soybean oil was measured [18], and the total phenols content found was 22.64 g GA/100 g sample, with antioxidant activity of 65 to 80% on DPPH. Regarding the antioxidant activity of soybean oil, the extract maintained a Peroxide Value close to 10 mEq/kg during the first 10 days of storage at 60°C, below the control, which was 12.5 mEq/kg. 

In another case, Romojaro and collaborators [19] studied the extract of *Q. ilex* subsp. *ballota* added to sunflower oil and orange juice. The extract presented a total phenol content of around 350 g acid gallic/100 g fw (fresh weight) and a total antioxidant activity (TEAC) of 2000 µmoles Trolox/100 g fw. The addition of the extract in the sunflower oil significantly decreased the lipid oxidation by 46.4% after heating it, while, in the orange juice, the extract positively impacted the sensory evaluation, presenting a general acceptance of 100.87% concerning the control. Likewise, Başyiğit and collaborators [17] evaluated the protective capacity of *Q. infectoria* gill extract in pasteurized milk, whose dominant compounds were ellagic acid (28,156.85 mg/kg dry) and catechins (716.21 mg/kg dry). The antioxidant activity of the extract was 2.29 and 1.65 mmol TEAC/g of DPPH and ABTS, respectively, also presenting a high antimicrobial activity against *Escherichia coli*. Lipid oxidation was not measured in this study.

Another important application of oak is the use of its wood to make barrels where different alcoholic beverages are aged. Phenolic compounds present in oak wood used for aging wines, rum, and other alcoholic beverages significantly impacted the flavor, aroma, and color of these beverages. Volatile phenols and benzoic aldehydes play an important role in contributing to the sensory characteristics of wines. Hydrolyzable tannins, such as ellagitannins, are particularly significant because they confer astringency and are involved in stabilizing pigment structures. Compounds like ellagic acid and ellagitannin impart complex flavors, spicy notes, and a characteristic brown color to wines and spirits aged in oak barrels [81]. Among the main compounds are hydroxybenzoic acids derived directly from benzoic acid, including gallic, gentisic, *p*-hydroxybenzoic, protocatechuic, syringic, salicylic, and vanillic acids and hydroxybenzoic aldehydes, such as syringaldehyde and vanillin, which are modified as aldehydes with carboxyl groups [82]. In addition, the antioxidant properties of these phenolic compounds and their free radical scavenging capacity help preserve alcoholic beverages during aging and improve their quality. In short, the phenolic compounds in oak wood are essential for producing high-quality alcoholic beverages with a unique and incomparable flavor [81]. Therefore, this evidence indicates the antioxidant potential of oak antioxidants when searching for anti-rancidity agents.

Despite the interest in using *Quercus* phenolic compounds as an antioxidant additive, very few foods have been tested. Most of them are focused on meat products, and the studies are carried out in combination with other preservation techniques (modified atmospheres). Therefore, it would be interesting to analyze its behavior in other food matrices highly susceptible to oxidation, such as oils and fried foods. Similarly, another area that has received less attention is the sensorial and toxicological profile of oak, which is critical if it is to be utilized extensively in food for human consumption.

The addition of oak extracts to food systems can have a significant impact on the odor and flavor of the food. In the case of extracts derived from oak wood, these contain compounds such as ellagitannins and volatile organic compounds, presenting aromas and flavors described as coconut, vanilla, nutty, and toasty [16,21]. In the case of extracts from oak leaves, it has been described that the high content of polyphenols can give astringent flavors [75]. When added to food systems, these compounds interact with food components such as fats and proteins, leading to changes in the food’s aroma and taste [83]. Research has shown that adding oak extracts can significantly alter the sensory profile of food. Soriano and collaborators [16] reported that when adding extract of *Q. alba* wood chips to pork patties, they acquired “woody” and “sweet spices” (clove and vanilla) odors, in addition to presenting a more intense coloration and positive acceptance. Meanwhile, when adding *Q. ilex* acorn extract to chicken patties, Ferreira and collaborators [15] reported a change in the color of the food, with greater consumer acceptance of both color and flavor. In the case of wines, Sindt and collaborators [84] reported that 3 compounds obtained from *Q. petraea* extract could be responsible for the bitter taste of wine, due to their prevalence during oak aging; meanwhile, by adding *Q. robur* extracts to wine, Jiménez-Moreno and collaborators [85] found that the wine exhibited more intense wood and spicy aromas after 18 months of bottle aging. These studies support *Quercus* as a promising ingredient in the food industry; however, special attention has to be paid to optimize the use of oak extracts to enhance the odor and flavor of food while ensuring food safety. 

In the case of oils, further research can be conducted to explore the potential uses and advantages of incorporating oak extracts into frying oils for fried foods. This can include controlled studies to determine the optimal amounts and combinations of oak extract for specific types of fried foods, as well as sensory analysis to evaluate the impact on flavor and odor. Additionally, research could focus on the stability and shelf life of the frying oils and the impact on the quality and taste of the fried foods over time. By conducting more research in this area, a deeper understanding of the benefits and limitations of using oak extracts in frying oils can be gained, providing valuable information for food manufacturers and industry professionals.

### 4.3. Toxicity of Oak Extracts

One of the issues faced by using oak extracts in food is their possible toxicity. Some studies have suggested that certain parts of the oak, mainly immature leaves, could present toxicity to terrestrial animals due to hydrolysable tannins and gallotannins [23]. Because of this, studies have been carried out to address this problem. The toxicity of *Q. crassifolia*, *Q. infectoria,* and *Q. sideroxyla* have been tested in rats, administered by subacute oral [74], acute via enema [86], and acute via gavage [87], respectively. The *Q. crassifolia* extract showed toxic effects at a repeatedly administered dose of 33 mg/kg bw/day and with an NOAEL (No Observed Adverse Effect Level) of 11 mg/kg bw/day, while the *Q. infectoria* extract dose of 10 g/kg showed no adverse effects on animal behavior, proposing that the maximum tolerated dose is above this value. In the case of *Q. sideroxyla*, the LD_50_ was determined at a dose greater than 5000 mg/kg, with no noticeable signs of adverse effects. Regarding in vitro studies, Pinto and collaborators [79] studied the cell viability and toxic effects of *Q. cerris* seeds used to prepare coffee-like beverages, finding the optimal range of non-cytotoxic concentrations in Caco-2 and HT29-MTX cell lines was between 0.1 and 1.0 μg/mL. Meanwhile, Hazwani and collaborators [88] tested the toxicity of a vaginal cream based on *Q. infectoria* in HeLa cells and female rats, finding moderate toxicity in the cells with IC50 values of 20.80 μg/mL, while the rats showed no adverse effects on their reproductive tract. 

Toxicological studies on foods added with oak extracts have not been carried out; however, in many places, infusions and fermented beverages of the leaves are consumed for medicinal purposes without apparent adverse effects [13,14,75]. The current knowledge on the toxicity of oak extracts is limited to studies performed on a cellular level and in rats. These studies’ results suggest that the toxicity of oak extract varies depending on the species and the method of administration. The few published studies show that oak extracts can have toxic effects in rats at high doses, while in vitro studies have found that oak extract has moderate toxicity in cells. Therefore, further toxicological studies on oak extract in food systems must be performed to determine its safety as an antioxidant ingredient. In addition, further studies are needed to determine if different extraction methods can generate safer extracts.

## 5. Conclusions

Oak extract has the potential to be used as a food antioxidant to reduce rancidity and improve the odor and flavor of food products. The use of oak extract has been studied in different forms, including infusions and fermented beverages, and its potential to be used as a frying oil additive have been shown. The results of these studies suggest that oak extract has antimicrobial, antiradical, and antioxidant properties that can help to prevent food rancidity and improve the overall quality of food products. However, there is a need for further research to fully understand the impact of oak extract on food systems and determine its safety for human consumption. Potential experiments that can be conducted to generate more knowledge include determining the efficacy of oak extracts to avoid rancidity in more food systems susceptible to this problem. It will also be important to evaluate the consumer acceptability of odor and flavor added by oak extract in food products, as well as its effectiveness as a frying oil additive in different food products. In addition, further studies could also focus on the optimal concentration and processing methods to maximize the beneficial effects of oak extract as a food antioxidant.

## Figures and Tables

**Table 1:** E number, maximum dosage range, and reported adverse reactions of some antioxidants used in the food industry.

Classification	Antioxidant Substance	Maximum Dosage Range (g/kg)	Treated Food	Reported Adverse Reactions	Reference
Natural	Ascorbic acid and its salts (E300–302)	0.2~5.0	Fresh peeled fruits and vegetables, wheat flour, fruit and vegetable products, fresh minced meat, charcuterie and salted products, fish, shellfish and mollusk unprocessed.	Not reported	[9,11]
	Tocopherols (E306) and their geometric isomers (E307–309 *)	Not reported	Meat, fish, nuts, vegetables, fruits, beverages and canned food, oils and emulsified fats, foods for infants and children.	Not reported	[11]
Synthetic	Propyl gallate (E310)	0.1~0.4	Canned nuts and seeds, gum-based candy, grilled meat, and noodles.	-Risk of methemoglobinemia. -Allergic reactions (eczema, hives, and stomach upset).	[9,11]
	Octyl gallate (E311)	0~0.0002	Lard, oils, fats for frying, fish oils and fat, sheep, poultry and beef, sauces, soups and broths dehydrated, pre-cooked cereals, spices and condiments, dehydrated granulated potatoes, chewing gum and snacks.	-Risk of methemoglobinemia. -Allergic reactions (eczema, hives, stomach upset, lichenoid lesions, cheilitis, and contact dermatitis).	[9,11,69,70]
	Tert-butylhydroquinone (TBHQ) (E319)	0.2	Moon cake, instant noodles, cookies, baked goods fillings.	-Carcinogenic at high doses.	[9,11]
	Butylhydroxyanisole (BHA) (E320)	0.2	Fats, oils, emulsified fat products, coarse grains, and instant noodles.	-Carcinogenic properties. -Risk of allergies (rash, chronic urticaria, angioedema, eczema, and contact dermatitis). -Increased levels of cholesterol and lipids in the blood. -Suspected hyperkinesis.	[9,11,69]
	Butylated hydroxytoluene (BHT) (E321)	0.2~0.4	Noodles, chewing gum-based candies, and air-dried aquatic products, among others.	-Carcinogenic properties. -Risk of allergies (rash, chronic urticaria, angioedema, eczema, and contact dermatitis). -Increased levels of cholesterol and lipids in the blood. -Suspected hyperkinesis.	[9,11,69]

* E307–E309 correspond to tocopherols of synthetic origin.

**Table 2:** Phenolic content, identified compounds, and antioxidant activity of various reported *Quercus* species.

Species	Raw Material/Extract Type	Phenolic Content		Phenolic Profile	Antioxidant Activity	References
		TPC	TFC			
*Quercus salicina*	Leaves/Ethanol	46.30 mg GAE/g	24.72 mg RE/g	Gallic acid, benzoic acid, ellagic acid, protocatechuic acid, chlorogenic acid, *p*-hydroxybenzoic acid, vanillic acid, ferulic acid, *p*-coumaric acid.	DPPH (IC_50_ mg/mL): Free: 0.067 Bound: 0.079 ABTS (IC_50_ mg/mL): Free: 0.523 Bound: 0.559	[77]
	Bark/Ethanol	35.89 mg GAE/g	1.41 mg RE/g	Protocatechuic acid, chlorogenic acid, syringic acid, vanillin, ellagic acid, cinnamic acid, ferulic acid, benzoic acid	DPPH (IC_50_ mg/mL): Free: 0.026 Bound: 0.565 ABTS (IC_50_ mg/mL): Free: 0.296 Bound: 2.796	
*Quercus crispula*	Leaves/Ethanol	12.25 mg GAE/g	4.18 mg RE/g	Chlorogenic acid, sinapic acid, *p-*coumaric acid, benzoic acid, ellagic acid	DPPH (IC_50_ mg/mL): Free: 0.100 Bound: 0.333 ABTS (IC_50_ mg/mL): Free: 1.008 Bound: 1.803	
	Bark/Ethanol	16.45 mg GAE/g	2.33 mg RE/g	Vanillin, ferulic acid, ellagic acid	DPPH (IC_50_ mg/mL): Free: 0.158 Bound: 0.446 ABTS (IC_50_ mg/mL): Free: 1.047 Bound: 2.074	
*Quercus serrata*	Leaves/Ethanol	25.97 mg GAE/g	28.18 mg RE/g	Chlorogenic acid, *p-*hydroxybenzoic acid, sinapic acid, benzoic acid, ellagic acid	DPPH (IC_50_ mg/mL): Free: 0.158 Bound: 0.446 ABTS (IC_50_ mg/mL): Free: 1.047 Bound: 2.074	
	Bark/Ethanol	16.15 mg GAE/g	2.49 mg RE/g	Protocatechuic acid, chlorogenic acid, syringic acid, vanillin, ferulic acid, benzoic acid, ellagic acid, cinnamic acid	DPPH (IC_50_ mg/mL): Free: 0.158 Bound: 0.446 ABTS (IC_50_ mg/mL): Free: 1.047 Bound: 2.074	
*Quercus coccifera*	Shell acorn/Ethanol	98.08 mg GAE/g	178.96 mg QE/g	Not reported	DPPH (Inhibition %): 82.35 FRAP (Absorbance at 700 nm): 1.86	[78]
	Acorn Cup/Ethanol	98.71 mg GAE/g	72.97 mg QE/g	Not reported	DPPH (Inhibition %): 91.09 FRAP (Absorbance at 700 nm): 2.04	
	Shelled Acorn/Ethanol	100.14 mg GAE/g	73.24 mg QE/g	Not reported	DPPH (Inhibition %): 88.58 FRAP (Absorbance at 700 nm): 1.52	
*Quercus cerris*	Seed/Water	2070.21 mg GAE/L	285.27 mg CAE/L	Ellagic acid, gallotannin, or ellagitannin derivatives	DPPH (IC_50_ μg mL^−1^): 271.61 FRAP (μg mL^−1^): 203.11 Scavenging capacities IC_50_ (μg mL^−1^): O_2_●^−^: 17.24 H_2_O_2_: 275.70 •NO: 0.65	[79]
*Quercus crassifolia*	Bark/Hot Water	860 mg GAE/g	43.6 mg QE/g	Not reported	Free radical scavenging (EC_50_ μg/mL) OH●: 467 O_2_●^−^: 58.1 H_2_O_2_: 22	[13]
	Bark/Ethanol	695 mg GAE/g	14.0 mg QE/g	Not reported	OH●: 2024 O_2_●^−^: 40.9 H_2_O_2_: 653	
*Quercus laurina*	Bark/Hot Water	474 mg GAE/g	24.1 mg QE/g	Not reported	Free radical scavenging (EC_50_ μg/mL) OH●: 1257 O_2_●^−^: 629 H_2_O_2_: 727	
	Bark/Ethanol	756 mg GAE/g	15.7 mg QE/g	Not reported	OH●: >4000 O_2_●^−^: 3213 H_2_O_2_: 519	
*Quercus scytophylla*	Bark/Hot Water	329 mg GAE/g	24.1 mg QE/g	Not reported	Free radical scavenging (EC_50_ μg/mL) OH●: 1865 O_2_●^−^: >4000 H_2_O_2_: 1102	
	Bark/Ethanol	521 mg GAE/g	12.9 mg QE/g	Not reported	OH●: >4000 O_2_●^−^: 406 H_2_O_2_: 1050	
*Quercus suber*	Leaves/Ethanol:Water (7:3)	10.6 mg GAE/g	8.2 mg C/g	Gallic acid, ellagic acid, chatechin, epicatechin, rutin, myricetin, quercetin	DPPH (Inhibition %): 22.7 ABTS (mg Trolox/g): 46.9 FRAP (mg Trolox/g): 54.5	[14]
*Quercus ilex*	Acorn/Food-Grade Acetone	928 mg GAE/100 g	Not reported	Not reported	DPPH (microM Trolox/g): 51.87	[15]
*Quercus alba*	Wood Chips/Aqueous Extract	2180.8 mg GAE/L	Not reported	Volatile compounds	DPPH (microM Trolox/L): 31.20 ABTS (microM Trolox/L): 32.00	[16]
*Quercus branti*	Acorn/Methanol	22.64 mg GAE/100 g	Not reported	Not Reported	DPPH (Inhibition %): ~77%	[18]
*Quercus robur*	Wood/Methanol	72.63 µg GAE/g	Not reported	Gallic acid, ellagic acid, protocatechuic acid, vanillic acid, 4-hydroxybenzoic acid, *p*-coumaric acid, sinapic acid, syringic acid, caffeic acid, ferulic acid, vanillin, protocatechuic aldehyde, syringaldehyde, coniferaldehyde, sinapaldehyde, scopoletin	FRAP (microM Trolox/mg): 0.82 ORAC (microM Trolox/mg): 0.92 ABTS (microM Trolox/mg): 1.59	[80]
*Quercus petraea*	Wood/Methanol	48.87 µg GAE/g	Not reported	Gallic acid, ellagic acid, protocatechuic acid, vanillic acid, *p*-coumaric acid, sinapic acid, syringic acid, caffeic acid, ferulic acid, vanillin, protocatechuic aldehyde, syringaldehyde, coniferaldehyde, sinapaldehyde, scopoletin	FRAP (microM Trolox/mg): 0.59 ORAC (microM Trolox/mg): 0.62 ABTS (microM Trolox/mg): 1.35	
*Quercus pyrenaica*	Wood/Methanol	41.48 µg GAE/g	Not reported	Gallic acid, ellagic acid, protocatechuic acid, vanillic acid, *p*-coumaric acid, sinapic acid, syringic acid, caffeic acid, ferulic acid, vanillin, protocatechuic aldehyde, syringaldehyde, coniferaldehyde, sinapaldehyde, scopoletin	FRAP (microM Trolox/mg): 0.54 ORAC (microM Trolox/mg): 0.65 ABTS (microM Trolox/mg): 1.19	

Free: free phenolics. Bound: bound phenolics. ~ indicates an approximate value. GAE: gallic acid equivalents. QE: quercetin equivalents. RE: rutin equivalents. CAE: catechin equivalents.

**Table 3:** Potential of oak extract to inhibit lipid oxidation (TBARS/Peroxide value) in different foods.

Species	Extracted Tissues	Added Food	Type of Extract (Solvent)	Lipid Oxidation (%/TBARS/mEQ/MDA)		Reference
				Extract	Control	
*Quercus* *suber*	Leaves	Chicken breast	Water Ethanol Water:Ethanol (1:1 *v*/*v*) Water:Ethanol (3:7 *v*/*v*)	95.3% 92.4% 97.2% 97.7%	95.9%	[14]
*Quercus ilex*	Acorn	Chicken patty	Food-grade acetone (60%)	~0.2 mg TBARS/kg	~0.8 mg TBARS/kg	[15]
*Quercus alba*	Wood chips	Pork patties	Aqueous extract	0.30 mg TBARS/kg (97.1%)	10.63 mg TBARS/kg	[16]
*Quercus branti*	Acorn	Soybean oil	Ethanol (95%)	~10 mEq/kg	~12.5 mEq/kg	[18]
*Quercus ilex* subsp. *Ballota*	Acorn	Sunflower oil	Liquid nitrogen	~3.5 mmol g^−1^ (46.4%)	6.53 mmol g^−1^	[19]

~ indicates an approximate value.

## Data Availability

All the data is contained within the article.
